# A Rapid Nano-Liquid Chromatographic Method for the Analysis of Cannabinoids in *Cannabis sativa* L. Extracts

**DOI:** 10.3390/molecules26071825

**Published:** 2021-03-24

**Authors:** Lucie Žampachová, Zeineb Aturki, Francesca Mariani, Petr Bednář

**Affiliations:** 1 Istituto per i Sistemi Biologici, Consiglio Nazionale delle Ricerche, Area della Ricerca di Roma I, Via Salaria Km 29.300, Monterotondo, 00015 Rome, Italy; luckazampachova@seznam.cz (L.Ž.); francesca.mariani@cnr.it (F.M.); 2 Department of Analytical Chemistry, Faculty of Science, Palacký University, 17. listopadu 12, 771 46 Olomouc, Czech Republic; petr.bednar@upol.cz

**Keywords:** cannabinoids, *Cannabis sativa* L., hemp inflorescences extracts, nano-LC-UV, nano-LC–MS

## Abstract

*Cannabis sativa* L. is an herbaceous plant belonging to the family of Cannabaceae. It is classified into three different chemotypes based on the different cannabinoids profile. In particular, fiber-type cannabis (hemp) is rich in cannabidiol (CBD) content. In the present work, a rapid nano liquid chromatographic method (nano-LC) was proposed for the determination of the main cannabinoids in *Cannabis sativa* L. (hemp) inflorescences belonging to different varieties. The nano-LC experiments were carried out in a 100 µm internal diameter capillary column packed with a C18 stationary phase for 15 cm with a mobile phase composed of ACN/H_2_O/formic acid, 80/19/1% (*v*/*v*/*v*). The reverse-phase nano-LC method allowed the complete separation of four standard cannabinoids in less than 12 min under isocratic elution mode. The nano-LC method coupled to ultraviolet (UV) detection was validated and applied to the quantification of the target analytes in cannabis extracts. The nano-LC system was also coupled to an electrospray ionization–mass spectrometry (ESI-MS) detector to confirm the identity of the cannabinoids present in hemp samples. For the extraction of the cannabinoids, three different approaches, including dynamic maceration (DM), ultrasound-assisted extraction (UAE), and an extraction procedure adapted from the *French Pharmacopeia*’s protocol on medicinal plants, were carried out, and the results achieved were compared.

## 1. Introduction

*Cannabis sativa,* or cannabis, belonging to the Cannabaceae family, is one of the world’s most widespread cultivations. It has been used for various purposes, including industrial textiles, food products (hemp), medical and psychotropic effects [1]. *Cannabis sativa* is characterized by a chemical complex composition with a wide number of compounds (about 500), including terpenes, flavonoids, stilbenoids, alkaloids, fatty acids and cannabinoids.

Over than 70 cannabinoids have been identified among which, Δ^9^-tetrahydrocannabinol (Δ^9^-THC), cannabidiol (CBD), cannabigerol (CBG), cannabichromene (CBC) and cannabinol (CBN) are the most abundant [2]. The taxonomic classification of this plant may result difficult due to its genetic variability. Recently, *Cannabis* has been divided into three main species based on the cannabinoid profile. Specifically, a fiber-type plant, *Cannabis sativa* L., namely also hemp or industrial hemp, which contains non-psychoactive cannabinoids. Hemp products can be used for textiles or food purposes and can be legally sold as tobacco substitutes, cosmetics and foodstuffs. A drug-type plant, called *Cannabis indica*, characterized by high levels of Δ^9^-THC, is used for medicinal or recreational purposes. The last one, *Cannabis ruderalis*, contains intermediate levels of the main components, such as CBD and Δ^9^-THC [3].

Nowadays, *Cannabis sativa* continues to be a controversial plant because it is considered as a drug of abuse for the presence of Δ^9^-THC. This psychoactive cannabinoid produces reactions, such as euphoria and relaxation, appetite stimulant, but it can cause serious side effects, such as cognitive deficits, anxiety, psychosis and addiction [4,5]. On the other hand, *Cannabis sativa*, owing to the presence of the non-psychoactive cannabidiol (CBD), possesses interesting therapeutic properties for the treatment of neuropathic pain, multiple sclerosis, nausea and vomiting due to chemotherapy, inflammatory diseases, epilepsy and glaucoma [6,7,8].

In *Cannabis*, cannabinoids are biosynthesized in the acidic form in plant tissues, psychologically inactive precursors, producing cannabidiolic acid (CBDA), and cannabigerolic acid (CBGA) in fiber-type plants, while Δ^9^-tetrahydrocannabinolic acid (Δ^9^-THCA) in drug-type ones. A spontaneous decarboxylation process can occur by heat or light, generating the neutral counterparts, including CBD, CBG and Δ^9^-THC [8]. In addition, to the properties of CBD and Δ^9^-THC described above, many studies proved that other minor components, such as CBG and CBC, possess anti-inflammatory, antibacterial and antifungal activities. Cannabinol (CBN) and cannabinolic acid (CBNA), deriving from the oxidative degradation products of Δ^9^-THC and Δ^9^-THCA, respectively, present in matured cannabis samples, have potent sedative properties [8,9,10].

Owing to the therapeutic effects of CBD, in recent years, many efforts have been directed to breeding CBD-rich strains, obtaining plants with CBD content up to 25% and less than 1% Δ^9^-THC [11]. Currently, the European Union (EU) lets the crop growing of hemp fiber-type varieties with Δ^9^-THC content below 0.2% for the legal sale of hemp products [12].

Therefore, the analysis of *Cannabis* is of utmost importance not only for forensic and legal purposes but has also become an important part of quality control of plant material used for food products or pharmaceuticals. Consequently, great efforts have been made to develop robust and sensitive analytical methods for the determination of cannabinoids in complex matrices, such as plants, foodstuffs, and biological samples. The most commonly used analytical methods are the chromatographic ones.

Gas chromatography (GC), hyphenated to flame ionization detection (FID) or mass spectrometry (MS), is a rapid and simple tool but presents the limitation that it provides the total content of cannabinoids. The heating of the sample in the injector port induces the decarboxylation of the cannabinoid acids converting them into the neutral form. Then, the determination of both acids and free cannabinoids requires a derivatization step, which is a time-consuming procedure and does not guarantee 100% of the yield [13,14].

HPLC has become the method of choice for determining cannabinoids in complex matrices because it allows the direct analysis of neutral and acidic cannabinoids, obtaining a complete chemical profile of the cannabis samples [14]. Almost all of the HPLC methods used for the analysis of cannabinoids have been developed based on reverse phase mode by using gradient elution.

HPLC and, more recently, ultrahigh-performance liquid chromatography (UHPLC) are coupled to ultraviolet (UV) and mass spectrometry (MS) detectors [15]. UV detector is the most used detection tool for the analysis of plant materials where the content of cannabinoids is quite high [16,17,18,19,20]. Mass spectrometry, due to its sensitivity and selectivity, remains the detection system of choice for the analysis of complex matrices, such as biological samples and food products [21,22,23,24].

In the last few decades, analytical research was focused on the development of miniaturized LC systems starting by decreasing the separation column diameter and the working flow.

Capillary and nano-liquid chromatography (CLC and nano-LC) provide various advantages over conventional HPLC, such as the wide reduction in mobile and stationary phase consumption, including toxic reagents; the small sample volume needed; high separation efficiency, the easy coupling to mass spectrometry (MS). In addition, the reduction of particle diameter and column length allows rapid analyses [25,26]. Currently, one of the most important advantages is the reduction of waste generation, in accordance with the principles of green chemistry [27].

Although the above-mentioned advantages and the good performance of nano-LC in several application fields [28,29], the use of this analytical tool for the analysis of cannabinoids in different matrices has not been widely exploited. To our knowledge, only two papers concerning the use of miniaturized chromatography for the analysis of cannabinoids, have been published. In particular, a nano-LC–UV method for the analysis of synthetic cannabinoids and Δ^9^-THC in herbal infusions was proposed [30]. More recently, an in-tube solid-phase microextraction (IT-SPME) coupled to a nano-LC system was developed for the detection of residues of *Cannabis* on different matrices, including herbal infusions, tobacco and some surfaces such as aluminum foil, office paper, cotton cloth [31].

**Figure 1:**
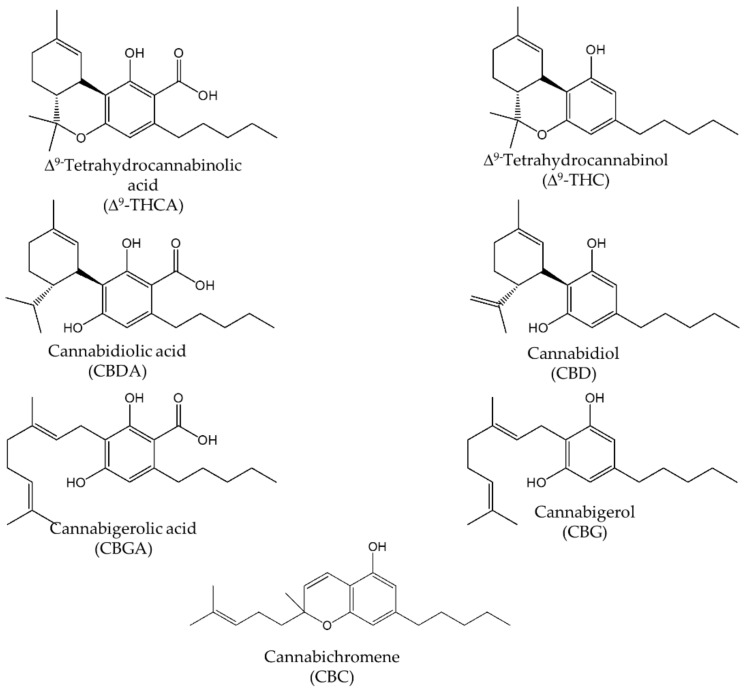
Chemical structures of main cannabinoids present in *Cannabis sativa* L.

Therefore, the aim of this work was the development and validation of a simple and rapid nano-LC-UV method for the simultaneous determination of the main cannabinoids, such as CBD, CBG, Δ^9^-THC and the corresponding acidic forms CBDA, CBGA, Δ^9^-THCA in hemp inflorescences of different varieties (for their chemical structures see Figure 1). For method optimization, several parameters, including the selection of the stationary phase, packed column length, mobile phase composition, and sample volume injection, were evaluated. The nano-LC-UV method was validated and applied for the determination of cannabinoids in hemp samples after an extraction procedure. The hyphenation of the nano-LC system with electrospray ionization–mass spectrometry (ESI-MS) detector, using an ion trap mass analyzer, allowed the identification of neutral and acidic cannabinoids present in the extracts. Concerning the sample preparation, three different extraction procedures, including dynamic maceration (DM), ultrasound-assisted extraction (UAE) and a hydro-alcoholic extraction procedure (HAE), were applied, and the results obtained were compared.

## 2. Results and Discussion

### 2.1. Nano-LC Method Development

#### 2.1.1. Selection of the Stationary Phase and Mobile Phase Composition

The studied cannabinoids CBD, CBG, CBC, Δ^9^-THC, are C21 terpenophenolic compounds obtained from the alkylation of alkyl resorcinol with a monoterpene unit [7]. They possess similar chemical structures differing in the alkyl chain or in several substituents, requiring then a highly efficient and selective analytical method for their resolution. In this work, a nano-LC method for the separation of the four selected cannabinoids was developed investigating some chromatographic parameters, such as the appropriate stationary phase, the mobile phase composition, sensitivity and suitable detectors (UV and MS). The optimized method was proposed as an alternative to conventional HPLC ones, where often a gradient elution mode is needed for the complete separation of the compounds and the costs of reagents and waste disposal are higher [16,23]. It is well-known that very fast analyses can be achieved employing UHPLC systems; however, this technique requires quite expensive instrumentation [32].

With the aim to obtain baseline resolution of all analytes in a reasonable time, three different RP stationary phases, i.e., Bidentate C18, (particle diameter, dp 4.2 µm, pore size, 100 Å, carbon load, 18%), ChromSpher C18, (particle diameter, dp 3 µm, pore size 100 Å, carbon load, 21%) and Gemini C18, (particle diameter dp 5 µm, pore size, 110 Å, carbon load, 14%) were investigated with a number of mobile phases, working in isocratic elution mode. Nano-LC experiments were carried out in capillary columns packed for 25 cm with these RP stationary phases. For each column, the composition of the mobile phase was varied in terms of organic modifier (ACN and/or MeOH) and the type and concentration of acid/buffer.

Bidentate C18 stationary phase, used in previous work for the separation of synthetic cannabinoids [30], was the first tested stationary phase, employing a mixture of ACN/H_2_O (80/20, *v*/*v*) containing 0.1% formic acid as mobile phase. In these experimental conditions, incomplete separation of the four cannabinoids was achieved due to less mutual resolution of CBG and CBD.

The ACN content in the mobile phase was varied in the range 70–85%, evaluating the influence on the resolution. As expected, by rising the ACN concentration, a general reduction of the retention times and a partial resolution of the two cannabinoids were observed. The best results in terms of resolution of the studied cannabinoids were obtained with an ACN content of 80% in the mobile phase.

Mixtures of MeOH/H_2_O in different content ratios (*v*/*v*) containing 0.1% formic acid were also tested, obtaining strong retention of the compounds with consequent broaden peaks.

Taking into account that in RP mode, MeOH generally improves the chromatographic selectivity, its addition in the range 5–10% to the mobile phase (ACN/H_2_O, 80/20, *v*/*v*), at the expense of either ACN or water, was investigated, observing a deterioration of resolution.

The effect of formic acid content in the range 0.1–2.0% was also studied, and the finest results were achieved using 1% concentration, with a mobile phase composed of ACN/H_2_O/formic acid, 80/19/1% (*v*/*v*/*v*).

The effect of buffer pH on retention and resolution was studied in the range 3.0–8.2. Ammonium formate and acetate buffers were prepared at the different pH values with a final concentration of 10 mM and added to the mixture ACN/H_2_O (80/20, *v*/*v*). It was noticed that an increase of the buffer pH provided a decrease of the retention of the compounds, with the co-elution of CBG and CBD without achieving any evident resolution improvement.

**Figure 2:**
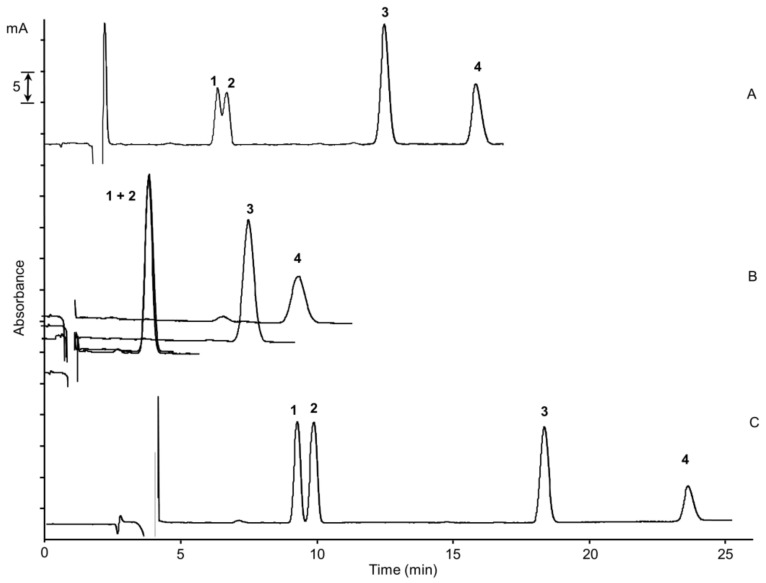
Nano-LC chromatograms of the separation of the four cannabinoids on different stationary phases: capillary columns (100 µm × 25 cm) packed with (**A**) Cogent Bidentate C18 dp 4.2 µm, (**B**) Gemini C18 dp 5 µm, (**C**) ChromSpher C18 dp 3 µm; mobile phase, acetonitrile/H_2_O/formic acid, 80/19/1% (*v*/*v*/*v*); UV detection, 214 nm; standard mixture concentration, cannabigerol (CBG) and cannabidiol (CBD) at the concentration of 15 µg/mL, cannabichromene (CBC) and Δ^9^-tetrahydrocannabinol (Δ^9^-THC) at a concentration of 30 µg/mL; injection time, 15 s; flow rate, 550 nL/min. (1) CBG, (2) CBD, (3) Δ^9^-THC, (4) CBC.

The chromatographic performance of another two reversed-phase columns, i.e., Gemini C18 and ChromSpher C18, was studied, varying the above-discussed parameters as well. The separation of the two cannabinoids CBG and CBD, with very close retention times, was not achieved under any of the experimental conditions tested with Gemini C18. On the other hand, ChromSpher C18 allowed the complete separation of the four cannabinoids within 25 min, employing a mixture composed of ACN/H_2_O/formic acid, 80/19/1% (*v*/*v*/*v*) as mobile phase. ChromSpher C18 column thus provides the best chromatographic performance among studied columns. This behavior can be explained with the highest carbon load of the ChromSpher C18 phase (21% with respect 18% and 14% of Bidentate C18 and Gemini C18, respectively) and a lower diameter particle (3 µm), increasing the chromatographic selectivity and the separation efficiency. In Figure 2, the comparison of the chromatographic separation of the four cannabinoids with the three different stationary phases was shown.

**Figure 3:**
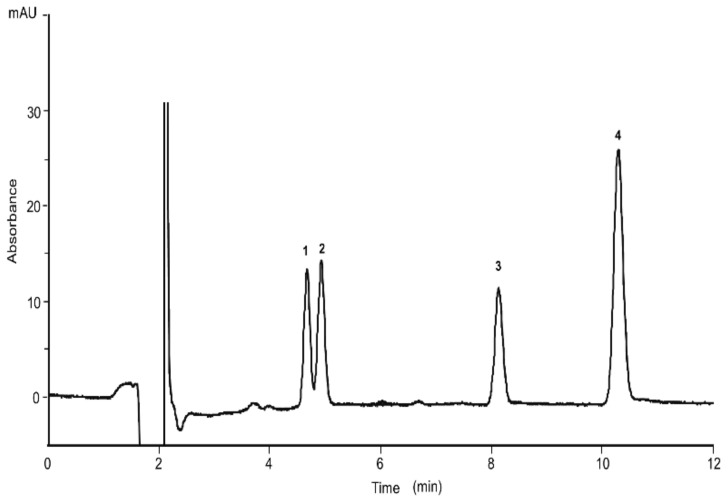
Separation of the four cannabinoids achieved with the short column: capillary column (100 µm × 15 cm) packed with ChromSpher C18 dp 3 µm; mobile phase, ACN/H_2_O/formic acid, 80/19/1% (*v*/*v*/*v*). UV detection, 214 nm; standard mixture concentration, CBG, CBD and Δ^9^-THC at the concentration of 15 µg/mL, CBC at a concentration of 25 µg/mL; injection time, 15 s; flow rate, 550 nL/min. (1) CBG, (2) CBD, (3) Δ^9^-THC, (4) CBC.

The effect of the column length shortening was studied as well. A column with a packed length of 15 cm also provided a baseline separation of the four analytes within 12 min. The nano-LC chromatogram of the four cannabinoids is depicted in Figure 3. All further experiments were carried with the short column.

#### 2.1.2. Sensitivity

The nano-LC system used for this study was equipped with a modified injection valve, which allowed the introduction of increasing sample volumes into the column. All the experiments were carried out using an injection time of 15 s corresponding to an injected volume of about 120 nL.

Since the use of capillary columns imposes the limit of introducing small volumes to increase the method sensitivity, an on-column focusing approach was adopted. The preconcentration method can be performed by selecting the appropriate sample solvent composition, allowing the injection of large sample volumes at the entrance of the column without compromising the chromatographic performance of the system. In fact, a dissolution solvent with a weak elution strength provides the focusing of the analyte zone [33]. Since the chromatographic separation of cannabinoids is based on a reversed-phase mechanism, mixtures with a high content of water were selected as the solvent because they offered the lowest eluotropic strength. A standard mixture of the cannabinoids at a final concentration of 1 µg/mL was prepared in water or H_2_O/ACN (90/10%, 80/20%, *v*/*v*). Selecting a solvent composed of water at the percentage of 100 and 90%, respectively, the broadening of CBC peak shape was observed. Hence, a mixture composed of H_2_O/ACN (80/20%, *v*/*v*) was selected as a solvent for sample dissolution in further experiments.

Analyses of the standard mixture with values of injection time in the range 30–240 s were carried out, plotting the injected volumes in the function of the peak height and peak width at half height (data not shown). A linear trend was obtained up to about 700 nL of volume injected (corresponding to an injection time of 180 s), while injections higher than 700 nL revealed an increase of band broadening with reduction of analytes’ resolution.

### 2.2. Evaluation of the Nano-LC Method in Terms of Precision, Linearity and Sensitivity.

Under the optimal conditions, the developed nano-LC method was investigated in terms of its repeatability, column-to-column reproducibility, linear dynamic range and sensitivity.

Due to the unavailability of the reference standards of the acidic forms of cannabinoids, their determination was performed considering the regression equations of the neutral forms allowing only a semiquantitative analysis of CBDA, CBGA and Δ^9^-THCA with lower accuracy.

Intra-day and inter-day repeatability, expressed as the average values and RSD%, for retention time (tr) and peak area (A), were calculated from the standard mixture analysis at the concentration of 15 µg/mL for CBG and CBD, 30 µg/mL for Δ^9^-THC and CBC, six times for a day (*n* = 6) as well as in different days (d = 3, *n* = 9). RSD% values for retention times ranged from 1.05–1.78 and 1.94–2.85 for intra-day and inter-day repeatability, respectively. Good results were also achieved for peak areas with RSD lower 3.71% and 4.36% for intra-day and inter-day experiments, respectively.

Column-to-column reproducibility was also evaluated, preparing three different capillary columns and testing them with the standard mixture of cannabinoids. Satisfactory results with RSD values lower than 3.96% for retention times and 5.01% for peak areas were obtained for all studied compounds. The results are shown in Table 1.

Sensitivity in terms of limits of detection (LOD) and quantification (LOQ) and the linear dynamic range were measured with an injection time of 30 s. LOD values, determined at a signal-to-noise ratio of 3 times, were obtained in the range 0.125–1.0 µg/mL. LOQ values were calculated at a signal-to-noise ratio of 10, and the values ranged between 0.5 and 2 µg/mL. Operating with an injection time of 180 s, an increment of the signal of about 5-fold was obtained, with a consequent decrease of the LOQ values in the range 0.1–0.5 µg/mL. Therefore, the found LOQ values were considered suitable for the aim of the work and quite comparable to those published in some works performed with HPLC and UPLC systems [16,18,32]. In addition, LOD and LOQ values with MS detection with an injection of 30 s were also estimated. It was found that the sensitivity data achieved with MS detection were lower than those measured with UV detection. In detail, LODs and LOQs (MS detection) were about 6–8 times and 9–11 times inferior, respectively, to the UV detection data.

Dynamic range was evaluated considering the concentration range between LOQ values and 100 or 150 µg/mL, selecting eight concentration levels for the calibration curves. For each concentration level, the injection of the standard mixture was repeated three times. The calibration curves were obtained, plotting the peak areas as a function of analyte concentration. The regression analysis was performed by calculating the coefficients of determination R^2^, which values > 0.9994, confirming good linearity.

The sensitivity and calibration data are reported in Table 2.

### 2.3. Analysis of Cannabinoids in Hemp Samples

#### 2.3.1. Extraction Procedures

As previously reported, the extraction procedure, as well as the solvent selection, can strongly influence the quantification of cannabinoids in hemp extracts. In our study, three different extraction procedures, including dynamic maceration (DM), ultrasound-assisted extraction (UAE) and a hydro-alcoholic extraction procedure (HAE) adapted from the protocol of French Pharmacopoeia on medicinal plants, were carried out, making a comparison between them. The first two approaches developed previously in the literature [19] based on the use of EtOH as green solvent resulted in being simple, fast and easy to carry out.

By comparing the two extraction procedures, DM proved to be the better approach for extracting the main cannabinoids in hemp samples in terms of recovery, in agreement with the data reported in [19].

The third hydro-alcoholic procedure, HAE, provided quite interesting results. When comparing the found amounts of cannabinoids with the two previous extractions, the latter approach showed in all the samples examined higher contents of CBDA and CBGA as well as CBD and CBG. This can probably be due to the longer extraction time. On the other hand, Δ^9^-THC and Δ^9^-THCA provided lower contents with respect to the amounts obtained with the other approaches in all the samples analyzed. This behavior could be due to the composition of the solvent mixture. A further investigation of this extraction approach will be performed in terms of extraction time and different ratios of the solvent mixture EtOH/H_2_O since hemp extracts free of psychotic cannabinoids, rich in CBDA and CBD obtained, could be used for nutraceutical purposes.

The accuracy of DM, UAE and HAE approaches (described in Section 3.4) was evaluated by using the recovery test. The extraction procedure was estimated fortifying one hemp sample with a standard mixture of CBD, CBG and Δ^9^-THC at two different concentration levels of 0.2 and 1.0 mg/g. In Table 3), the recovery values for the extraction procedures are depicted. As mentioned before, the DM procedure showed a slightly higher accuracy with recovery data ranged between 80–95%, while UAE showed values of recovery in the range 75–91%. With the HAE procedure, the highest recovery values were achieved for CBD and CBG (90–104%), but the lowest values of recovery for Δ^9^-THC were found. All the recovery data are depicted in Table 3).

#### 2.3.2. Determination of Main Cannabinoids in Hemp Extracts

The developed nano-LC-UV method was applied for the analysis of the main cannabinoids in several cannabis extracts. Since the standards of CBDA, CBGA and Δ^9^-THCA were not available, the determination of the main cannabinoids in hemp extracts was made possible by combining the information arising from the coupling of the nano-LC system with both UV and MS detectors. The MS experiments were performed in positive ion mode, obtaining a good ionization also for the acidic cannabinoids. In Table 4, the retention times and MS data of the main cannabinoids present in hemp extracts are reported. As we can observe in Table 4, the retention times achieved with the nano-LC–MS system are approximately two minutes longer than the times obtained with the nano-LC-UV system. In the hyphenation with MS, it is necessary to consider the total length of the capillary column, which is oriented in front of the MS by means of a tip capillary for an entire length of 22 cm. Instead, in the nano-LC-UV coupling, the column was used with an effective length of 16 cm corresponding to the detector cell. Then, it is necessary to take into account the 6 cm after the window cell. The length of the column was not modified in order to be used in both systems. Concerning the MS data, CBD and Δ^9^-THC, as well as CBDA and Δ^9^-THCA have the same molecular masses, leading to the same precursor ions [M + H]^+^ with values of 315.5 and 359.3 for the neutral and acidic forms, respectively. Thus, their discrimination and quantification were achieved by the combination of the mass spectrometer with nano-LC separation. CBG and CBGA were easily distinguished from the other main cannabinoids due to their different molecular masses (*m*/*z* values of precursors were 317.5 and 361.3, respectively). From the nano-LC–MS chromatograms, the main cannabinoids eluted with the following elution order, CBDA, CBGA, CBG, CBD, Δ^9^-THC, CBC, Δ^9^-THCA in accordance with the data reported in the literature [16].

Hemp inflorescences were subjected to three different extraction methods (for the extraction procedures, see Section 3.4) and analyzed with the optimized nano-LC-UV method. The estimated amounts of cannabinoids, expressed as mg/g (dry weight), found in eight different hemp inflorescences analyzed are reported in Table 5. The data reported in the table confirm that the cannabinoid profile was strongly dependent on the genetic variability, type of cultivations and pedoclimatic conditions [1].

As expected, being C1-C5 fiber-type samples, the main compounds present in almost all analyzed samples were CBDA and CBD. CBDA was the most abundant cannabinoid in samples C2–C5 varying its content in the range 23.8–40.9 mg/g. The second main compound was CBD, the decarboxylated form of the acidic precursor. Its content was between 5.9 and 32.5 mg/g. The samples showed a characteristic cannabinoid profile of fresh plant material. The values found were in accordance with the data reported in the literature [19,24]. In sample C1, on the other hand, CBDA and CBD showed an opposite profile where CBD was the most abundant. This difference in the C1 sample may be due to the material obtained from an old plant or inappropriate storage conditions where the material has been exposed to light or heat, leading to the decarboxylation of the acid form of the cannabinoid [7]. Samples C4, C5, C2, subjected to the extraction with the HAE procedure, showed a different profile where both CBDA and CBD were present at almost a 1:1 ratio, in accordance with the results of Brighenti et al. [19].

From the achieved results, it appears that extraction procedures, storage conditions, and stability play a crucial role in the quality of hemp inflorescences.

C6 and C7 samples showed high contents of CBGA with values of 37.5 and 31.8 mg/g, whereas CBG was present with amounts of 10.9 and 2.3 mg/g, respectively. The determined amounts of CBG and CBGA were in agreement with the data reported in the literature [33]. Owing to the documented therapeutic properties of CBG, recently, large-scale legal production of this chemotype has been encouraged developing CBG-rich cannabis strains where CBG concentration was increased to levels of over 15%.

In all fiber-type samples analyzed (C1–C7), except C3, the content of Δ^9^-THC was found to be lower than the LOQ limit with an estimated value under the legal limit of 0.2%. The content of Δ^9^-THC in all samples was confirmed with GC experiments previously performed in a certified laboratory. Owing to the very low content of the psychotropic cannabinoid and the high amount of CBDA, these extracts could be utilized for pharmaceutical or nutraceutical purposes.

Sample C8 was identified as a drug-type *Cannabis* sample for the amount of Δ^9^-THC, particularly due to the contents of CBD and THC present, this sample could be better classified as recreational *Cannabis* [24].

**Figure 4:**
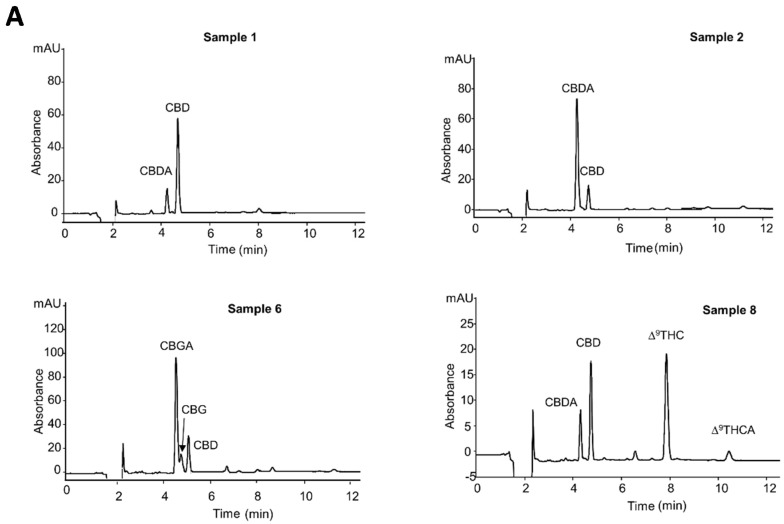

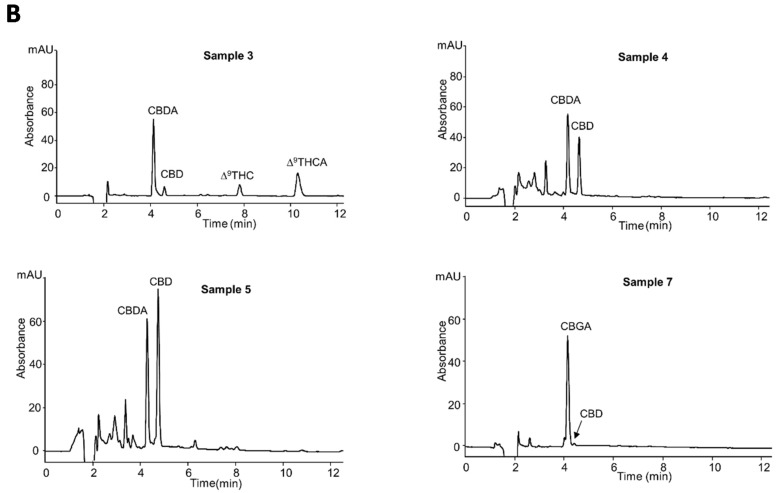
(**A**,**B**) Nano-LC-UV profiles of the hemp extracts. Experimental conditions: capillary columns (100 µm × 15 cm) packed with ChromSpher C18 dp 3 µm; mobile phase, ACN/H_2_O/formic acid, 80/19/1% (*v*/*v*/*v*). UV detection, 214 nm; injection time, 30 s; flow rate, 550. The samples here reported, were subjected to a dynamic maceration (DM) extraction procedure, with the exception of samples 4 and 5, which were subjected to the hydro-alcoholic extraction procedure (HAE).

In Figure 4A,B the nano-LC-UV chromatograms of the analyzed hemp extracts are shown.

**Figure 5:**
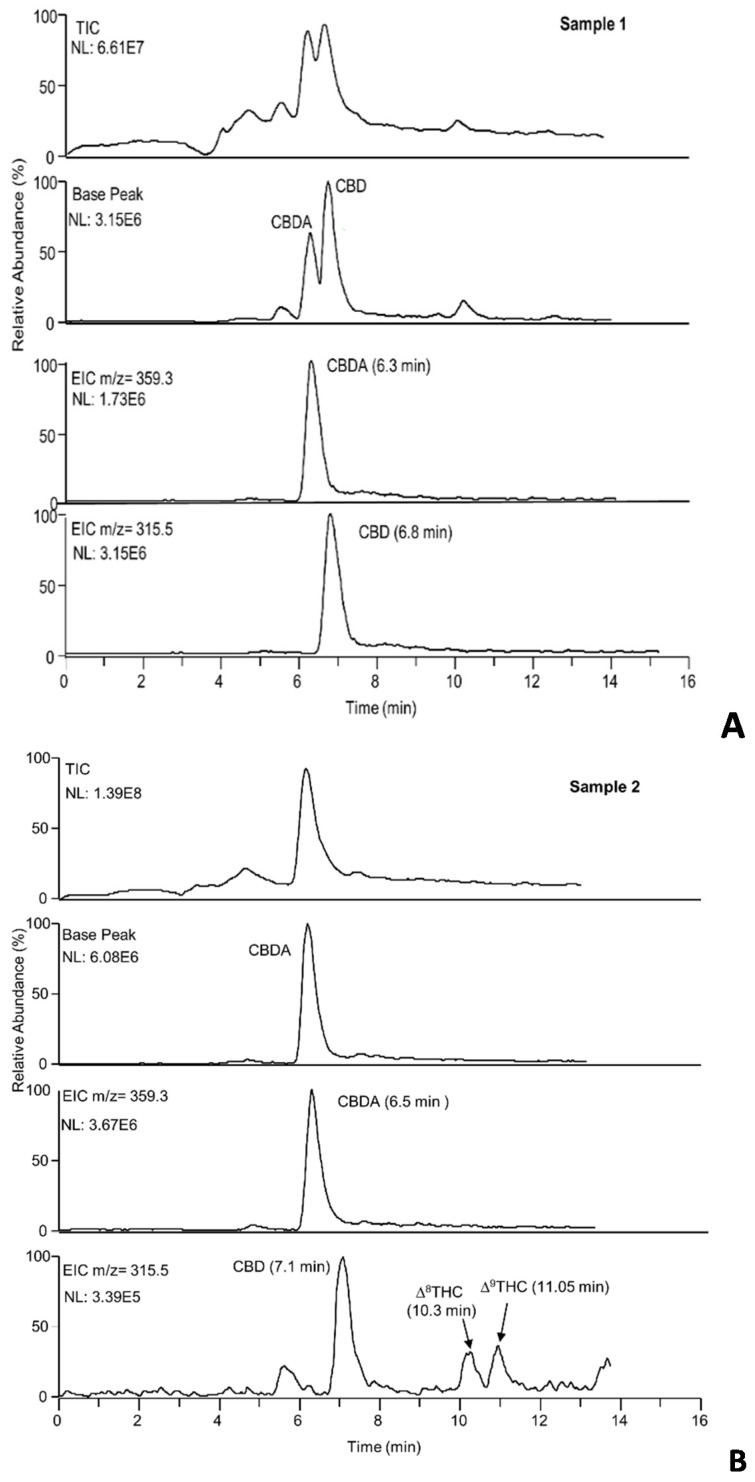

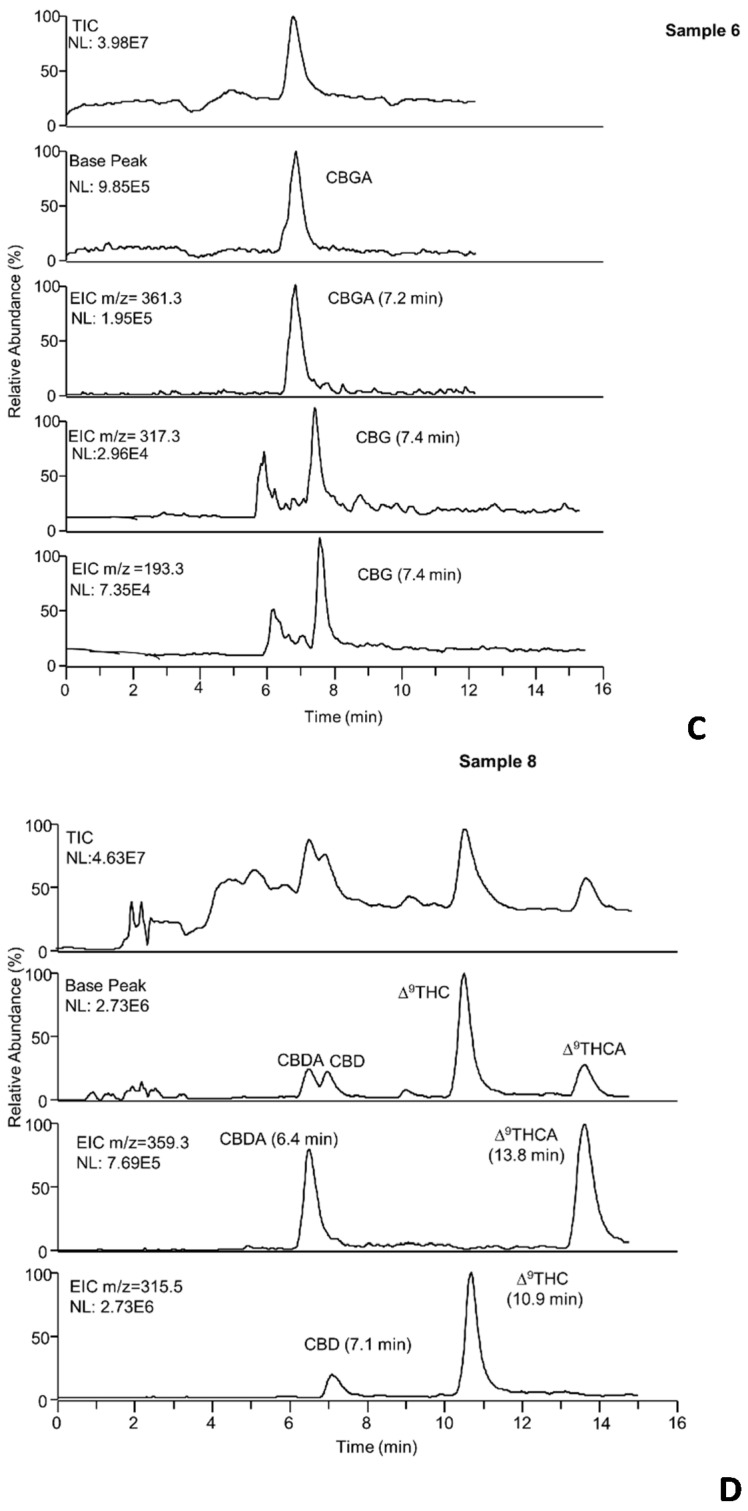
(**A**–**D**) Total ion, base peak and extracted ion chromatograms of the hemp extracts. Experimental conditions: capillary column, packed with ChromSpher C18 for 15 cm, effective length 22 cm, mobile phase, ACN/H_2_O/formic acid, 80/19/1% (*v*/*v*/*v*); injection time, 30 s; flow rate, 550 nL/min. For MS parameters, see Section 3.2. The hemp extracts analyzed were subjected to a DM extraction procedure.

In Figure 5A–D, the total ion (TIC), base peak, and extracted ion chromatograms of samples 1, 2, 6, 8 are depicted. As can be observed, the base peak and the extracted ion chromatograms (EIC) allow identifying the main cannabinoids, including also Δ^9^-THC and Δ^9^-THCA not quantified with the nano-LC-UV system in samples (C1, C2, C6).

Samples C1 and C2 were mainly characterized by CBDA and CBD with relative abundances that confirm the amounts calculated with the nano-LC-UV method. In C2, two different peaks with the same molecular masses of 315.3 were observed. From the data reported in the literature and the retention times obtained, we assumed that the two peaks were probably the two isomers Δ^8^-THC and Δ^9^-THC.

C6 confirmed mainly the presence of CBGA. A very weak signal for the precursor ion (317.3 *m*/*z*) of CBG was observed, while the product ion (193.3 *m*/*z*) was more intense.

In C8, Δ^9^-THC and Δ^9^-THCA were the cannabinoids most abundant. This was confirmed by MS signals more intense with respect to the CBD and CBDA ones.

Figure S5E–H concerning the total ion base peak and extracted ion chromatograms of samples 3, 4, 5, 7, as well as the MS spectra of all samples analyzed are reported and described in the Supplemental Materials.

## 3. Materials and Methods

### 3.1. Chemicals and Reagents

All chemicals were of analytical reagent grade and were used without further purification. Formic and acetic acid were purchased from Carlo Erba (Milan, Italy). Acetonitrile (ACN), methanol (MeOH) and ultrapure double-distilled water were obtained from VWR (International PBI, Milan, Italy). Cannabigerol (CBG), cannabichromene (CBC), cannabidiol (CBD) were purchased from NCLABS s.r.o., (Prague, Czech Republic). Stock standard solutions (1 mg/mL) were prepared by dissolving each analyte in MeOH. A standard solution of 1 mg/mL of Δ^9^-tetrahydrocannabinol (Δ^9^-THC) in methanol was obtained from Cerilliant (Round Rock, TX, USA). All standard cannabinoids were stored at −20 °C. To perform the analyses, the cannabinoids mixture was daily prepared, diluting the standard solutions with the mobile phase at the desired concentration. Mobile phases were daily obtained by mixing ultrapure water, organic solvents as ACN or MeOH and suitable amounts of acid additive in 10 mL volumetric flasks.

### 3.2. Instrumentation

Nano-LC experiments were carried out with a laboratory assembled instrumentation employing a Spectra System P2000 conventional gradient HPLC pump, a UV-vis on-column detector, Spectra Focus PC1000 (both from Thermo Separation Products, San Jose, CA, USA), and a modified injection valve equipped with an external loop of 50 µL (Enantiosep GmbH, Münster, Germany). Detection wavelengths were set at 210–214 nm, and data were collected using ClarityTM Advanced Chromatography Software (Data Apex Ltd., Prague, Czech Republic).

To reduce the flow rate from µ- to nL/min, the HPLC pump, delivering MeOH in continuous and the injector were connected to a passive split-flow system. For this purpose, both the HPLC pump and injection valve were joined to a stainless steel T-piece (Vici, Valco, Houston, TX, USA) by means of 500 µm id stainless steel tubes with lengths of 50 and 5 cm, respectively. The third entrance of the T-piece was connected to the MeOH reservoir of the pump through a fused silica capillary (50 µm id × 50 cm), achieving continuous recycling of the organic solvent. To minimize the dead volume and consequently reduce the band broadening effect, the capillary column was directly inserted into the modified injector, which was used for both sample loading and mobile phase reservoir. Samples and mobile phases were introduced into the capillary column through the injection valve by filling the loop with the sample solutions, switching the valve for the appropriate time and then flushing the loop with the mobile phase. The analysis started immediately after positioning the injector device in the injection mode. When the mobile phase had to be changed, it was directly introduced into the modified injector, significantly reducing the consumption of organic solvents [34]. The flow rate was estimated by connecting a microsyringe of 10 µL (Hamilton, Reno, NV, USA) to the capillary column end through a Teflon tube and by measuring the volume of mobile phase collected for 5 min.

In the optimized conditions, the flow rate of the column was about 550 nL/min. A standard mixture composed of CBG and CBD at the concentration of 15 µg/mL, CBC and Δ^9^-THC at a concentration of 30 µg/mL was injected for 15 s (corresponding to an injected volume of 80 nL at a flow rate of 550 nL/min).

The qualitative analysis of the different cannabinoids in hemp samples was performed by hyphenating the nano-LC system with an MS detector, LCQTM ion-trap (Thermo Finnigan San Jose, CA, USA) by means of a laboratory assembled nano-spray-ESI interface [35]. The device was assembled as follows: the end of the chromatographic column was joined to the fused silica tip emitter (25 µm id × 375 µm od × 10.5 cm) throughout a stainless steel union with a zero dead volume (VICI-VALCO Instruments, Houston, USA). An external power supply (CZE1000 R, Spellman High Voltage Electronics, NY, USA) was connected to the union, applying an external voltage. The interface was positioned on an XYZ translation stage in order to align the emitter tip with the MS orifice (distance 3 mm). The correct position was controlled by an analogical-video system. The emitters were laboratory prepared to obtain the tip shape by using a very simple rotating disk supporting fine emery paper.

The electrospray mass spectrometry measurements were carried out in a-positive ionization mode acquiring the MS spectra in full scan mode in the range 150–400 *m*/*z*. In order to optimize the MS parameters, the MS tune was carried out in automatic mode infusing CBD at a concentration of 0.1 µg/mL diluted in mobile phase with a flow rate of 1 µL/min. The compounds were detected applying the following conditions: spray voltage, 1.8 kV; capillary voltage, 34 V; capillary temperature, 190 °C.

### 3.3. Capillary Column Preparation

Nano-LC experiments were performed in uncoated fused silica capillaries (100 µm id × 375 µm od × 40 cm) from Composite Metal Services, Hallow, UK), packed in our laboratory following the slurry packing procedure as described previously [36]. For preliminary studies, three different stationary phases were considered, including Type-C Silica CogentTM Bidentate C18, 4.2 µm particle size (MicroSolv Technology Corporation, Eatontown, NJ, USA); ChromSpher C18, 3 µm particle size (Varian, Palo Alto, CA, USA); Gemini C18, 5 µm particle size from (Phenomenex, Torrance, CA, USA).

Briefly, one end of the capillary was connected to a temporary mechanical frit (VICI Valco, Houston, TX, USA) to retain the packing material and the other end to a stainless steel HPLC pre-column (10 cm × 4.1 mm i.d.), which was used as a reservoir for the slurry. Packing material (about 20 mg) suspended in acetone was packed into the capillary using an LC pump (PerkinElmer series 10 LC, Palo Alto, CA, USA) for a length of about 35.0 cm. Afterward, the column was flushed for 30 min with water to remove the packing solvent from the capillary, followed by a 5 mM NaCl solution for 10 min. The inlet and outlet frits were prepared to sinter the particles for 8 s at 700 °C using a laboratory-made heating wire, flushing the capillary continuously with water. The temporary frit was removed, and the excess packing material was eliminated by flushing with water. The detection window was prepared at 2 cm from the outlet removing the polyimide layer with a razor. For the nano-LC experiments, capillary columns packed for 15 and 25 cm different lengths were prepared.

### 3.4. Sample Preparation

The fiber-type cannabis inflorescences, belonging to different varieties, were kindly provided by Mr. Valerio Rosati and his local farm, located in Montelibretti, Rome, Italy. The hemp inflorescences were subjected to three different extraction procedures described below.

#### 3.4.1. Dynamic Maceration (DM)

The hemp inflorescence sample was weighed (0.25 g) and crushed into powder in a mortar. 10 mL of EtOH as extraction solvent was added, and the mixture was introduced in 15 mL glass tubes. The extraction was performed at room temperature for 15 min under magnetic stirring. The solution was then filtered through a paper filter, and the residue was extracted with the same procedure twice with 10 and 5 mL of EtOH, respectively. All filtrates were combined and brought to the final volume of 25 mL with the extraction solvent in a volumetric flask. The extract was centrifuged at 7000 rpm (RCF = 3831.3 g), diluted ten times with a mixture of water/ACN 80/20% (*v*/*v*) and injected into the nano-LC system.

#### 3.4.2. Ultrasound-Assisted Extraction (UAE)

Similar to the DM procedure, a 0.25 g hemp sample was mixed with 10 mL of EtOH and inserted into an ultrasound bath thermostated at 40 °C for 15 min. The solution was then filtered through a paper filter. The residue was extracted again twice with 10 and 5 mL of EtOH, respectively. The three filtrates were combined and adjusted to the final volume of 25 mL with the extraction solvent in a volumetric flask. The extract was centrifuged at 7000 rpm (RCF = 3831.3 g), diluted ten times with a mixture of water/ACN 80/20% (*v*/*v*) and injected into the nano-LC system.

#### 3.4.3. Hydro-Alcoholic Extraction Procedure (HAE).

This extraction procedure followed the French Pharmacopeia draft monograph for medicinal plants and was slightly modified [37]. Two grams of grounded hemp sample were mixed with 20 mL of an aqueous solution of EtOH at 60%. The solution was kept in the dark at room temperature for 15 days. Then, the extract was filtered with sterile gauze. The volume of the filtrate was adjusted with the aqueous solution of EtOH to a final volume of 20 mL. The extract was centrifuged at 7000 rpm (RCF = 3831.3 g) for 10 min, diluted ten times with a mixture of water/ACN 80/20% (*v*/*v*) and injected into the nano-LC system.

## 4. Conclusions

In this work, a rapid nano-LC method for the determination of the major cannabinoids present in hemp inflorescences was developed. A laboratory-assembled nano-LC system was used for chromatographic experiments. A capillary column with a length of 15 cm provided complete separation of all analytes using isocratic elution in less than 12 min. The nano-LC method developed for the separation of cannabinoids was found to be fast and more selective than other methods optimized using other advanced separation techniques, such as HPLC and UHPLC, where a gradient elution is needed. This can lead to saving analysis time since the column does not require a conditioning time between the chromatographic runs.

Simple sample treatment based on the use of EtOH and water, not requiring then hazardous solvents were applied, comparing the different extraction procedures.

In our study, the HAE procedure shows some interesting results concerning obtaining samples with great contents of phytocannabinoids and low amounts of Δ^9^-THC, particularly suitable for nutraceutical purposes.

The developed nano-LC-UV method was investigated in terms of precision, linearity and sensitivity showing satisfactory results. The coupling of the nano-LC system with the MS detector provided the identification of the major cannabinoids (CBDA, CBGA, Δ^9^-THCA, CBD, CBG, Δ^9^-THC). The developed method allows profiling the hemp inflorescences extracts and thus controls their quality. It can also be useful in establishing the cannabinoid profile of cannabis varieties and in discriminating between fiber-type and drug-type *Cannabis* samples. In addition, the distinction between the different chemotypes can provide valuable information concerning therapeutic and nutraceutical effects. The positive results reached in this study proved that the nano-LC method developed could be proposed as a valuable approach for the determination of cannabinoids profiles in hemp extracts. A great advantage deriving from using a low flow rate, resulting in a minimal consumption of reagents and samples, makes this analytical system more cost-effective and eco-friendlier with respect to the conventional chromatographic tools.

## Figures and Tables

**Table 1:** Intra-day, inter-day repeatability and column reproducibility expressed as average values and RSD% for retention times (*tr*) and peak areas (*A*).

Analytes	Intra-Day Precision (*n* = 6)		Inter-Day Precision (3 Days, *n* = 15)		Column-to-Column Reproducibility (3 Columns, *n* = 9)	
	*tr*, min (RSD%)	Peak Area (*A*) (RSD%)	*tr*, min (RSD%)	Peak Area (*A*) (RSD%)	*tr*, min (RSD%)	Peak Area (*A*) (RSD%)
CBG	4.75 (1.05)	49.41 (3.71)	4.88 (1.94)	45.78 (4.20)	4.82 (3.46)	47.94 (4.55)
CBD	4.94 (1.24)	47.44 (2.76)	5.01 (2.85)	48.44 (3.76)	5.12 (3.78)	48.23 (3.88)
Δ^9^THC	8.08 (1.56)	43.04 (2.98)	8.25 (2.04)	42.48 (3.95)	8.28 (3.96)	43.76 (4.22)
CBC	10.27 (1.78)	37.50 (3.38)	10.35 (2.50)	39.48 (4.36)	10.31 (3.65)	41.78 (5.01)

**Table 2:** Sensitivity and calibration data of the nano-LC-UV method.

Analytes	UV Detection		MS Detection		Linear Dynamic Range (μg/mL)	Slope ± SD	Intercept ± SD	*R^2^*
	LOD (µg/mL)	LOQ(µg /mL)	LOD (µg/mL)	LOQ (µg/mL)				
CBG	0.125	0.50	0.020	0.055	1–100	14.537 ± 0.038	−33.574 ± 2.856	0.9998
CBD	0.125	0.50	0.020	0.055	0.5–100	14.861 ± 0.047	−18.298 ± 1.785	0.9996
Δ^9^THC	0.25	0.50	0.035	0.055	0.5–100	19.285 ± 0.076	−32.452 ± 2.129	0.9994
CBC	1.0	2.0	0.125	0.175	2–150	6.488 ± 0.029	−27.255 ± 1.985	0.9998

**Table 3:** Mean recovery data with the three extraction procedures.

Extraction Procedure	Initial Content (mg/g ± SD)	Content Found after Addition (mg/g ± SD)		Recovery (%) (RSD, %)	
DM		Spiked Level (0.2 mg/g)	Spiked Level (1 mg/g)	Spiked Level (0.2 mg/g)	Spiked Level (1 mg/g)
CBG	n.d.	0.17 ± 1.8	0.88± 3.5	85. 1 (4.9)	88.0 (3.4)
CBD	7.9 ± 1.4	6.8 ± 0.9	7.5 ± 1.5	86.1 (5.1)	94.9 (2.9)
Δ^9^-THC	n.d.	0.16 ± 4.8	0.92 ± 2.9	80.0 (3.2)	92.0 (1.8)
**UAE**					
CBG	n.d.	0.15 ± 4.4	0.85 ± 2.9	75.0 (3.8)	85.0 (5.7)
CBD	7.6 ± 2.3	6.1 ± 3.7	6.9 ± 2.1	80.2 (4.9)	90.7 (2.8)
Δ^9^-THC	n.d.	0.15 ± 1.9	0.91 ± 5.1	75.0 (4.9)	91.0 (3.1)
**HAE**					
CBG	n.d.	0.18 ± 4.9	1.04 ± 2.5	90.0 (1.8)	104.0 (4.1)
CBD	8.1 ± 1.2	7.6 ± 2.5	8.0 ± 3.8	93.8 (2.5)	98.7 (1.3)
Δ^9^-THC	n.d.	0.13 ± 5.8	0.76 ± 2.4	65.0 (4.7)	76.0 (4.1)

**Table 4:** Retention times with nano-LC–MS, MS data of the major cannabinoids detected in hemp extracts.

Peak Number	Compound	*tr* (min)	MW	Precursor ion (*m/z*) [M + H]^+^	Product Ion (*m/z*)
1	CBDA	6.4	358.5	359.3	341.5
2	CBGA	6.7	360.5	361.3	343.5
3	CBG	6.9	316.5	317.5	193.3
4	CBD	7.2	314.5	315.5	-
5	Δ^9^THC	11.1	314.5	315.5	-
6	CBC	11.9	314.5	315.5	-
7	Δ^9^THCA	13.2	358.5	359.3	341.5

**Table 5:** Cannabinoids content in hemp inflorescences ethanolic extracts.

Sample Type	Sample No.	Content (mg/g ± SD)					
Fiber-Type *Cannabis*		CBDA	CBGA	CBG	CBD	Δ^9^THC	Δ^9^THCA
1	1-UAE	4.7 ± 0.1	-	-	16.1 ± 0.1	-	-
	1-DM	5.6 ± 0.1	-	-	23.1 ± 0.3	-	-
	1-HAE	7.2 ± 0.6	-	-	30.5 ± 4.6	-	-
2	2-UAE	28.1 ± 0.6	-	-	5.9 ± 0.2	<LOQ **	<LOQ **
	2-DM	31.7 ± 0.6	-	-	6.6 ± 0.4	<LOQ **	<LOQ **
	2-HAE	33.9 ± 0.2	-	-	22.7 ± 0.9	-	-
3	3-UAE	31.7 ± 1.1	-	-	6.4 ± 1.4	0.8 ± 1.9	10.6 ± 1.4
	3-DM	35.3 ± 1.8	-	-	7.9 ± 0.2	1.2 ± 1.1	12.4 ± 0.2
	3-HAE	40.9 ± 0.7	-	-	32.5 ± 1.7	<LOQ	<LOQ
4 *	4-HAE	23.8 ± 0.5	-	-	19.5 ± 1.3	-	-
5 *	5-HAE	27.4 ± 1.7	-	-	31.3 ± 0.9	-	-
6	6-UAE	-	36.3 ± 1.8	9.7 ± 1.8	13.8 ± 0.5	-	-
	6-DM	-	37.5 ± 0.9	10.9 ± 1.9	10.4 ± 1.1	-	-
	6-HAE	-	39.5 ± 1.3	12.7 ± 0.8	15.3 ± 0.3	-	-
7 *	7-UAE	-	27.9 ± 0.7	1.6 ± 0.5	-	-	-
	7-DM	-	31.8 ± 0.1	2.3 ± 0.3	-	-	-
Drug-type *Cannabis* 8	8-UAE 8-DM	4.1 ± 0.8 5.2 ± 1.1	- -	- -	7.7 ± 1.4 9.1 ± 1.7	11.8 ± 0.3 13.3 ± 0.7	1.8 ± 0.3 3.2 ± 0.7
	8-HAE	7.8 ± 1.4	-	-	10.4 ± 1.7	6.5 ± 1.4	<LOQ *
							

* 4 and 5 samples were subjected only to HAE procedure; * 7 was subjected to DM and UAE approaches; ** Δ^9^THC and Δ^9^THCA identified by nano-LC–MS, but not quantified by nano-LC-UV.

## Data Availability

The data presented in this study are available in this article.

## Supplementary Materials

The following are available online, Figure S5E–H: title: Total ion, base peak and extracted ion chromatograms of the hemp extracts. Figure S6: MS spectra of the hemp extracts.

## Sample Availability

No availability.
