# Supplementary material for: A Rapid Nano-Liquid Chromatographic Method for the Analysis of Cannabinoids in Cannabis sativa L. Extracts
Source: Molecules. 2021 Mar 24;26(7):1825. doi: 10.3390/molecules26071825 (PMC8036269; doi:10.3390/molecules26071825)
Supplement: Supplementary file 1 [file molecules-26-01825-s001.pdf]

Supporting Information Section

In this section Figures 5 E, H, F, G of samples 3, 4, 5, 7, are reported and discussed.

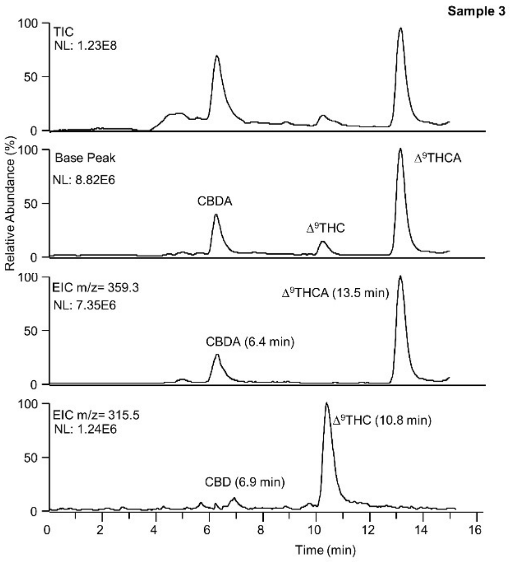

Figure 5 E

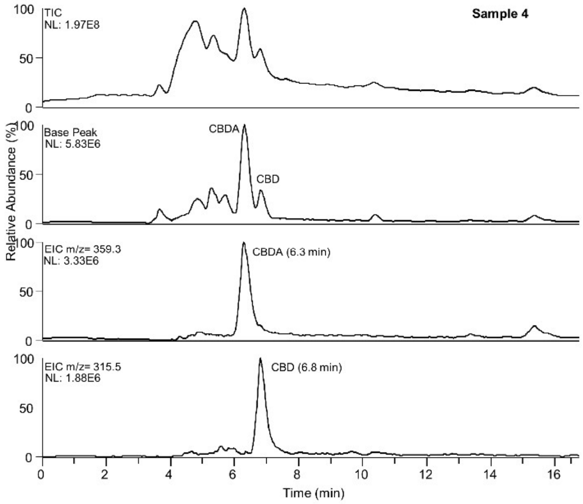

Figure 5 F

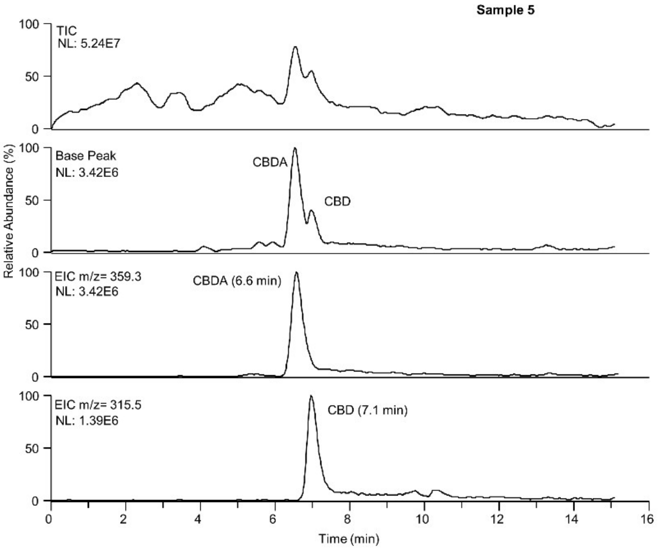

Figure 5 G

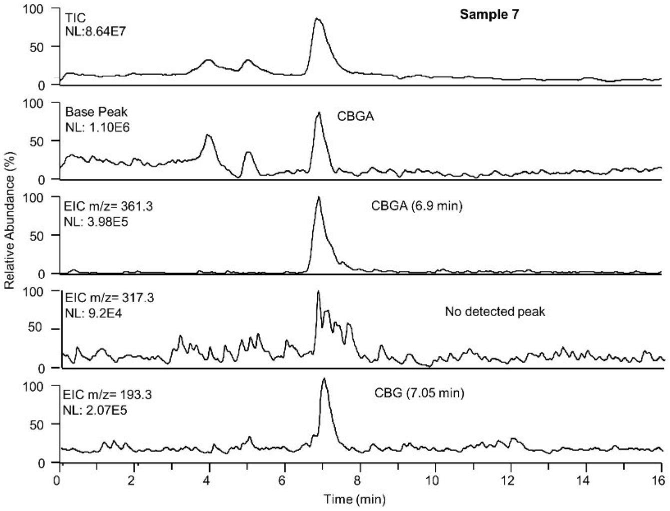

Figure 5 H

Figure 5. E, F,G,H: Total ion, base peak and extracted ion chromatograms of the hemp extracts. Experimental conditions: capillary column, packed with Chromspher C18 for 15 cm, effective length 22 cm, mobile phase, ACN/H2O/formic acid, 80/19/1 % (v/v/v); injection time, 30 s; flow rate, 550 nL/min. For MS parameters see section 3.2. Samples C 3 and C7 were subjected to a DM extraction procedure while C4 and C5 to the HAE approach.

The MS chromatogram of sample C3 (Fig. E) showed unusual contents of Δ^9-THC and Δ^9-THCA confirming the data found by analysing the sample with the nano-LC-UV system. Samples C4 and C5, subjected to the HAE extraction procedure, showed contents of CBDA and CBD at almost a ratio of 1:1, that was confirmed by the intensity of the signal of the precursor ions of CBDA and CBD (shown in Fig. 5 F and 5 G). Sample C7 as well as C6 showed a high amount of CBGA which MS signal was acceptable while a very low signal was observed for CBD.

The MS spectra of all the samples are shown below:

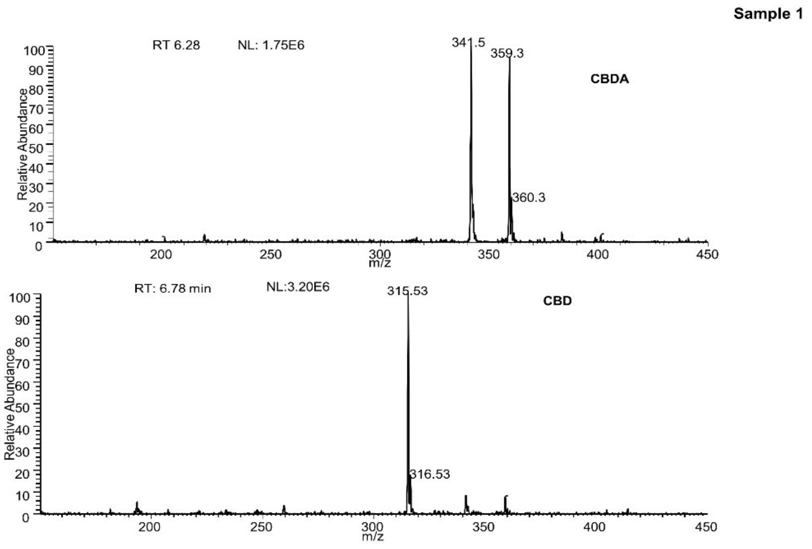

Figure MS 1

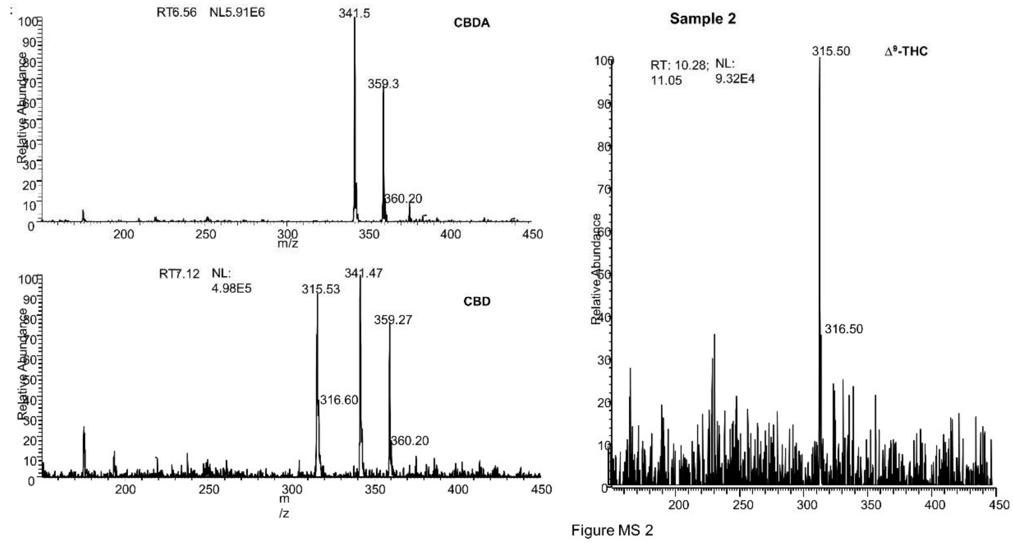

Figure MS 2

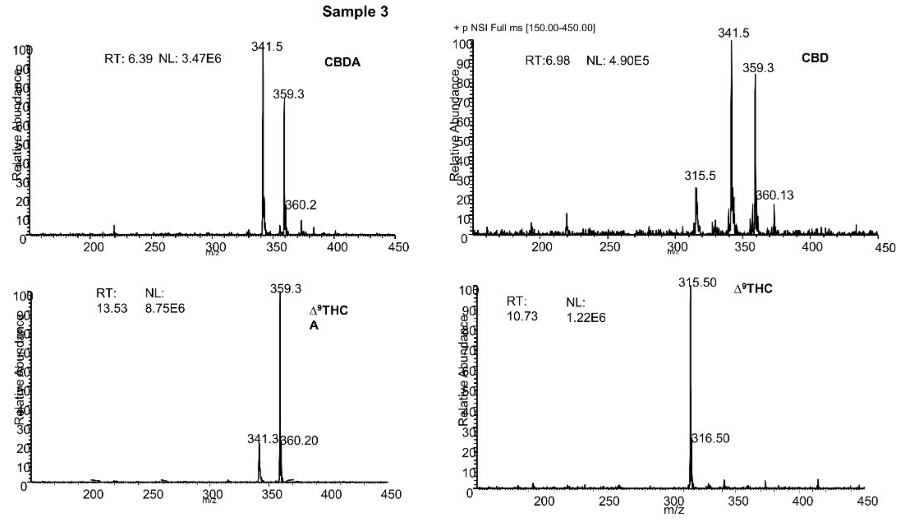

Figure MS 3

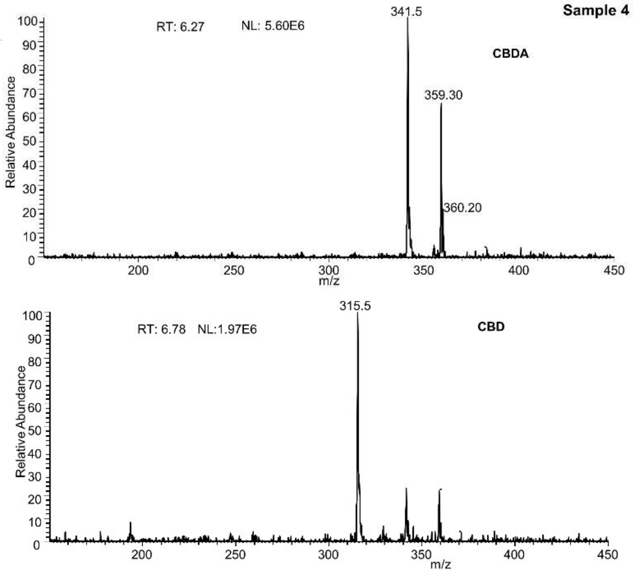

Figure MS 4

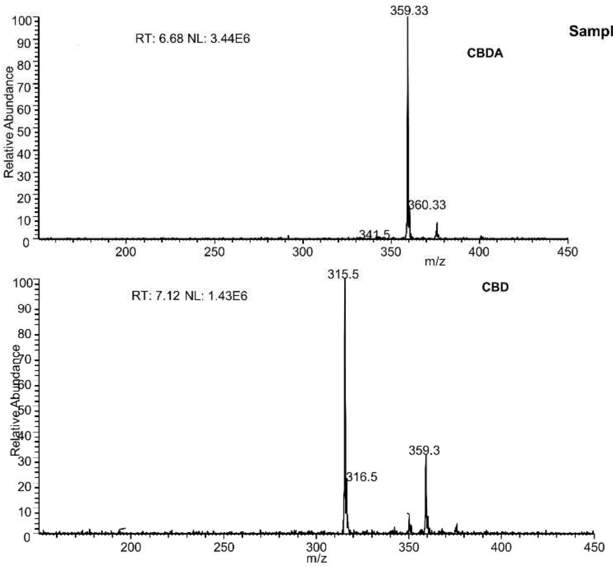

Figure MS 5

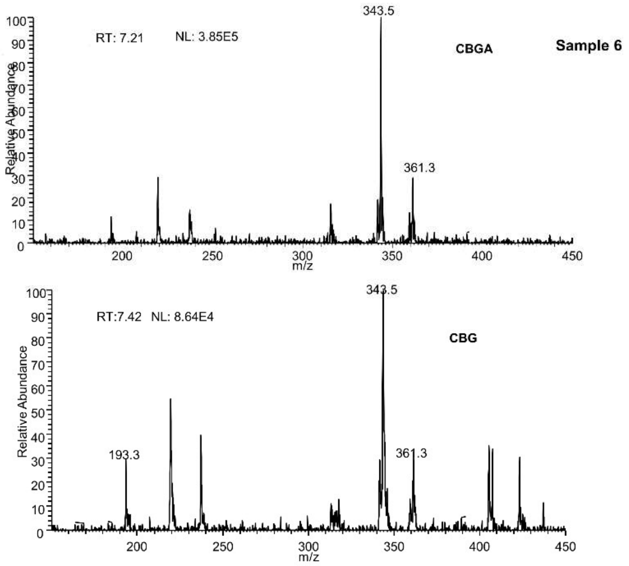

Figure MS 6

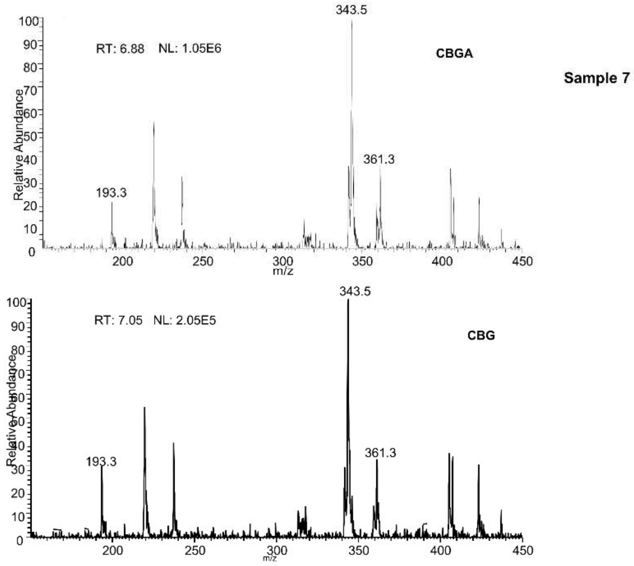

Figure MS 7

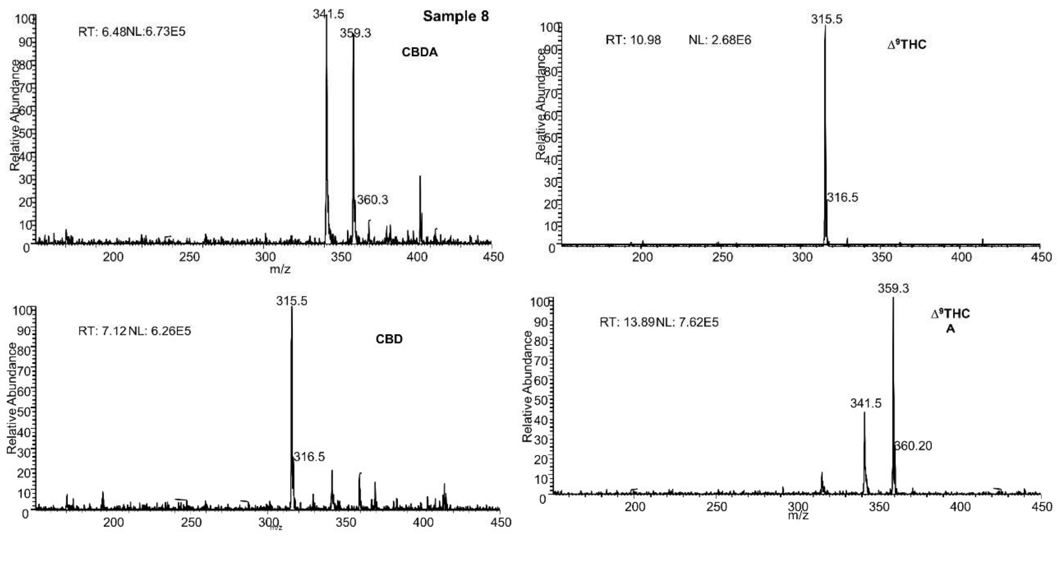

Figure MS 8

Figures MS 1 – 8: For chromatographic and MS parameters see Figures 4 A and B and Figures 5
